# Supplementary material for: Noncoding function of super enhancer derived mRNA in modulating neighboring gene expression and TAD interaction
Source: bioRxiv. 2023 Dec 5:2023.12.05.570115. Preprint. [Version 1] doi: 10.1101/2023.12.05.570115 (PMC10723268; doi:10.1101/2023.12.05.570115)

bioRxiv preprint doi: https://doi.org/10.1101/2023.12.05.570115; this version posted December 5, 2023. The copyright holder for this preprint (which was not certified by peer review) is the author/funder. This article is a US Government work. It is not subject to copyright under 17 USC 105 and is also made available for use under a CC0 license.

Supplementary Figure 1 | *CpoxeRNA* tissue expression, *Cpox* TSS deletion, and primer location. **A.** Tissue expression pattern of *CpoxeRNA* (*Gm41464*). Data from NCBI. **B.** Gel image for *Cpox* TSS KO confirmation. **C**. *Cpox* mRNA expression level after TSS Knock out. EV, empty vector. Data are mean ± s.d.; four biological replicates. Unpaired two-tailed t-test. **D.** Sanger sequencing result for *Cpox* TSS KO, black line shows the deletion site. **E**. Location of primers for *Dcbld2* and *St3gal6* qRT-PCR. UCSC genome browser tracks show the isoforms of *Dcbld2* and *St3gal6*. Two primer pairs each used to detect *Dcbld2* and *St3gal6* are heighted in yellow. TAD boundary region is highlighted in grey. **F.** Expression level of *Dcbld2-1* and *Dcbld2-2* in undifferentiated (UMEL) and differentiated (iMEL) MEL cells. Data are mean ± s.d.; three biological replicates. Unpaired one-tailed t-test. **G.** 5kb resolution HiC map shows the *Dcbld2-Cpox* TAD in undifferentiated and differentiated Fetal Liver cell, data from Bi et al.(39) Black arrow shows the TAD corner loop.

Supplementary Figure 2 | *Cpox* knock down does not affect erythropoiesis. A. CD71/Ter119 flow-cytometric result of differentiated shCTRL, shCPOX-4, shCPOX-5 cells. **B.** shCTRL, shCPOX-4, shCPOX-5 cells differentiated 5 days with 2% DMSO. **C.** qRT-PCR result shows nascent RNA expression level in shCTRL and shCPOX-4 UMEL cells. Two biological replicates. Data are mean ± s.d., unpaired one-tailed t-test. **D.** Insulation score and TAD boundary data from G1ER cell cycle in situ HiC experiment shows *Dcbld2* and *Cpox* overlap with TAD boundary. CTCF and RAD21 ChIP-seq data from asynchronized G1ER cell from the same study as G1ER cell cycle in situ HiC experiment were shown below.

Supplementary Figure 3 | Transcription at TAD boundary correlates with TAD insulation strength. A. Upper panel shows the insulation score, TAD boundary, and CTCF position in G1ER and MEL cells. Lower panel shows the DRIP-qPCR result of R loop formation at the TAD boundaries after *Cpox* mRNA knock down in UMEL cells. Position of primers are highlighted in the upper panel. Data are mean ± s.d.; unpaired one-tailed t-test with three technical replicates. **B.** HiC matrix shows the *Dcbld2-Cpox* TAD in G1ER and CH12.LX cells. Differential matrix was shown below, TAD

bioRxiv preprint doi: https://doi.org/10.1101/2023.12.05.570115; this version posted December 5, 2023. The copyright holder for this preprint (which was not certified by peer review) is the author/funder. This article is a US Government work. It is not subject to copyright under 17 USC 105 and is also made available for use under a CC0 license.

boundary loop was highlighted with circle in the differential matrix. **C.** Fold change of *Cpox* and *Dcbld2* expression level in G1ER and CH12.LX cell. Expression data from ENCODE RNA-seq data.

Supplementary Figure 4 | ChIP-qPCR primer location and Sanger sequencing of genetic deletion of *Cpox* intron 5 TFBS. A. Location of primers used for ChIP-qPCR in Figure 3 C and D. **B.** *Cpox* intron 5 TFBS KO gel image. **C.** *Cpox* intron 5 TFBS KO BLAST result, MEL p300 ChIP seq data from ENCODE. **D.** Sanger sequencing data, black line shows the deletion site.

Supplementary Figure 5 | *Cpox* intron5 TFBS deletion activates intra-TAD enhancer and TAD boundary gene *Dcbld2*. A. Western blot result shows CPOX protein level in WT and *Cpox* intron 5 TFBS depleted cells. **B.** qRT-PCR result shows the expression of *Cpox*, *St3gal6*-1, and *Dcbld2*-1 after *Cpox* intron 5 TFBS deletion in UMEL cells. Three biological replicates. Data are mean ± s.d., unpaired one-tailed t-test.

Supplementary Figure 6 | Distribution of TAD boundary gene interaction pattern and expression. A. Bar plot shows the distribution of different types of interaction observed at TAD boundary in GM12878, CH12.LX, and G1ER cells. **B.** Expression level at each TAD boundary for Promoter-Terminator (PT), Promoter-Genebody (PG) and Promoter-Promoter (PP) boundary pairs in GM12878, CH12.LX, and G1ER cells. Dots inside of the violin plots show the mean value. Kruskal-Wallis test, P values ≤ 0.05 indicating a significant difference.

Supplementary Figure 7 | TAD boundary gene and TSS mutations. A. K562 HiC maps(8,34) of *EEFSEC-RPN1*, *AP2S1-ARHGAP35*, *IGF2BP2-TRA2B* pairs, insulation score and TAD boundary are shown above the HiC matrix. Names of oncogenes are in red. **B**. Oncoplots shown the landscape of ICGC mutations located in the promoters of TAD anchor non-oncogene-oncogene pairs, *PSMD3-CDK12* and *RPN1-EEFSEC*.

Supplementary Fig. 1

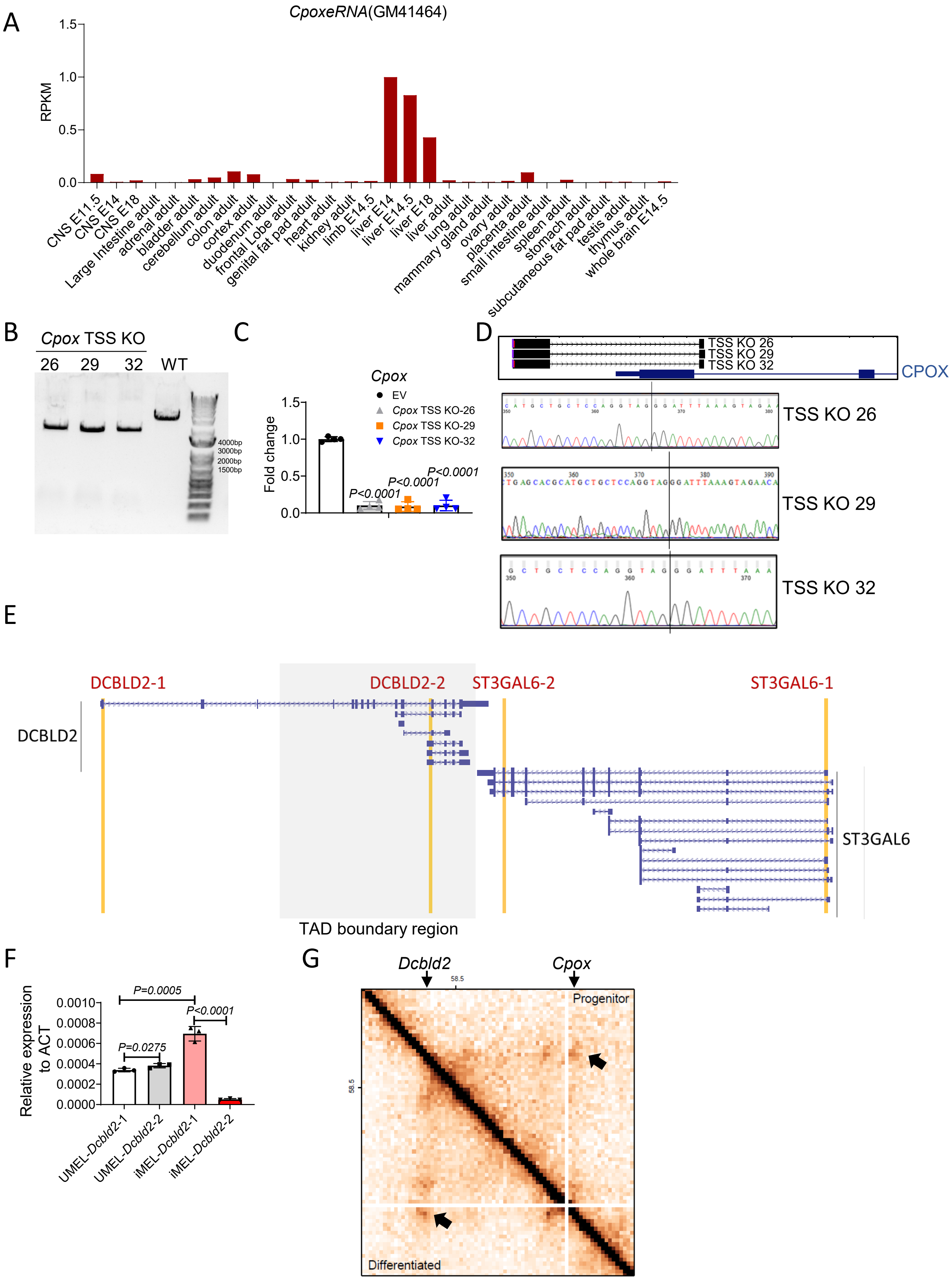

Supplementary Fig. 2

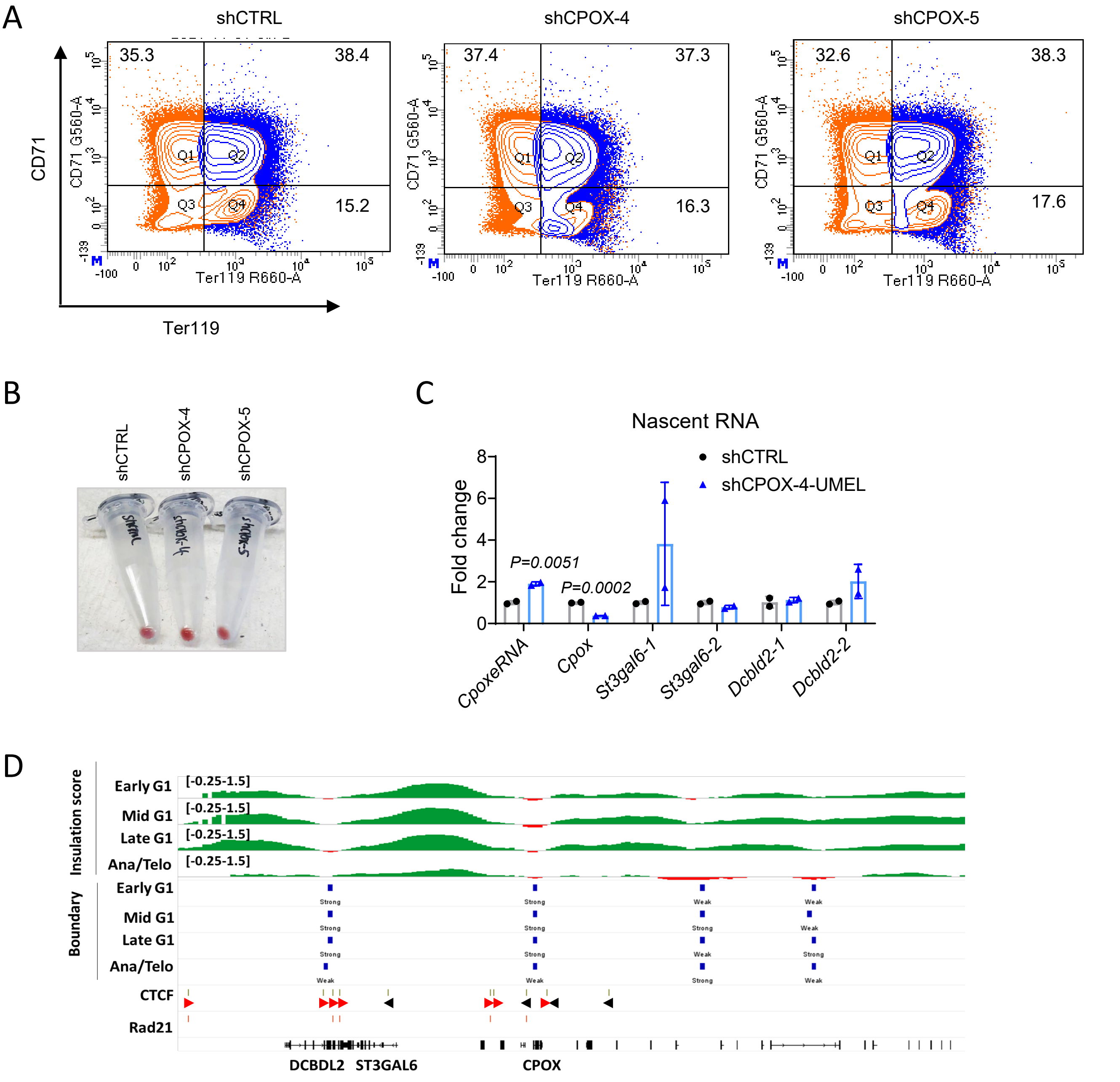

Supplementary Fig. 3

A

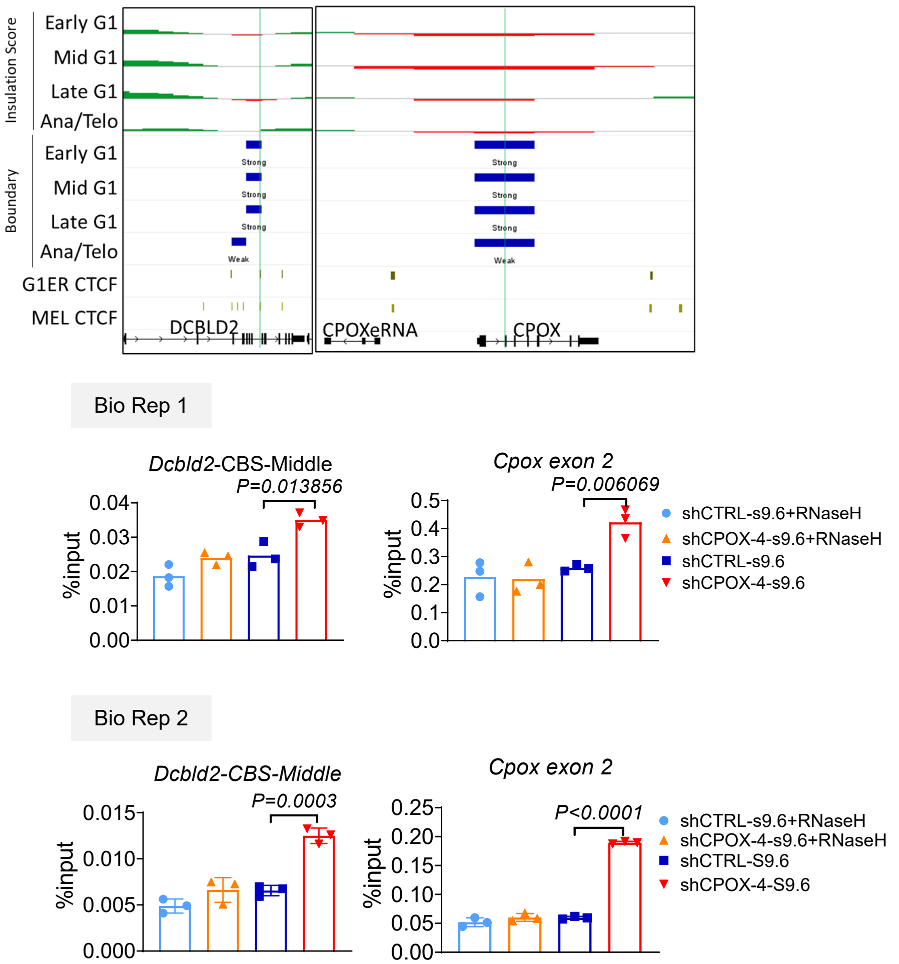

B

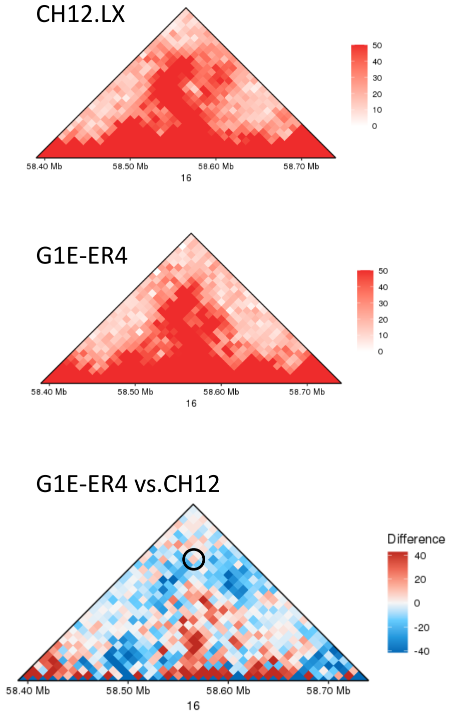

C

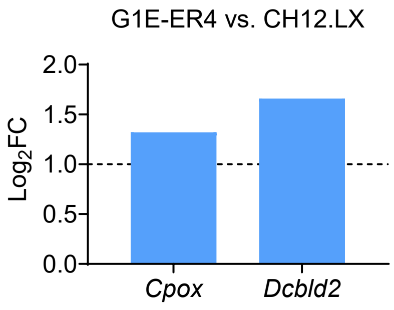

Supplementary Fig. 4

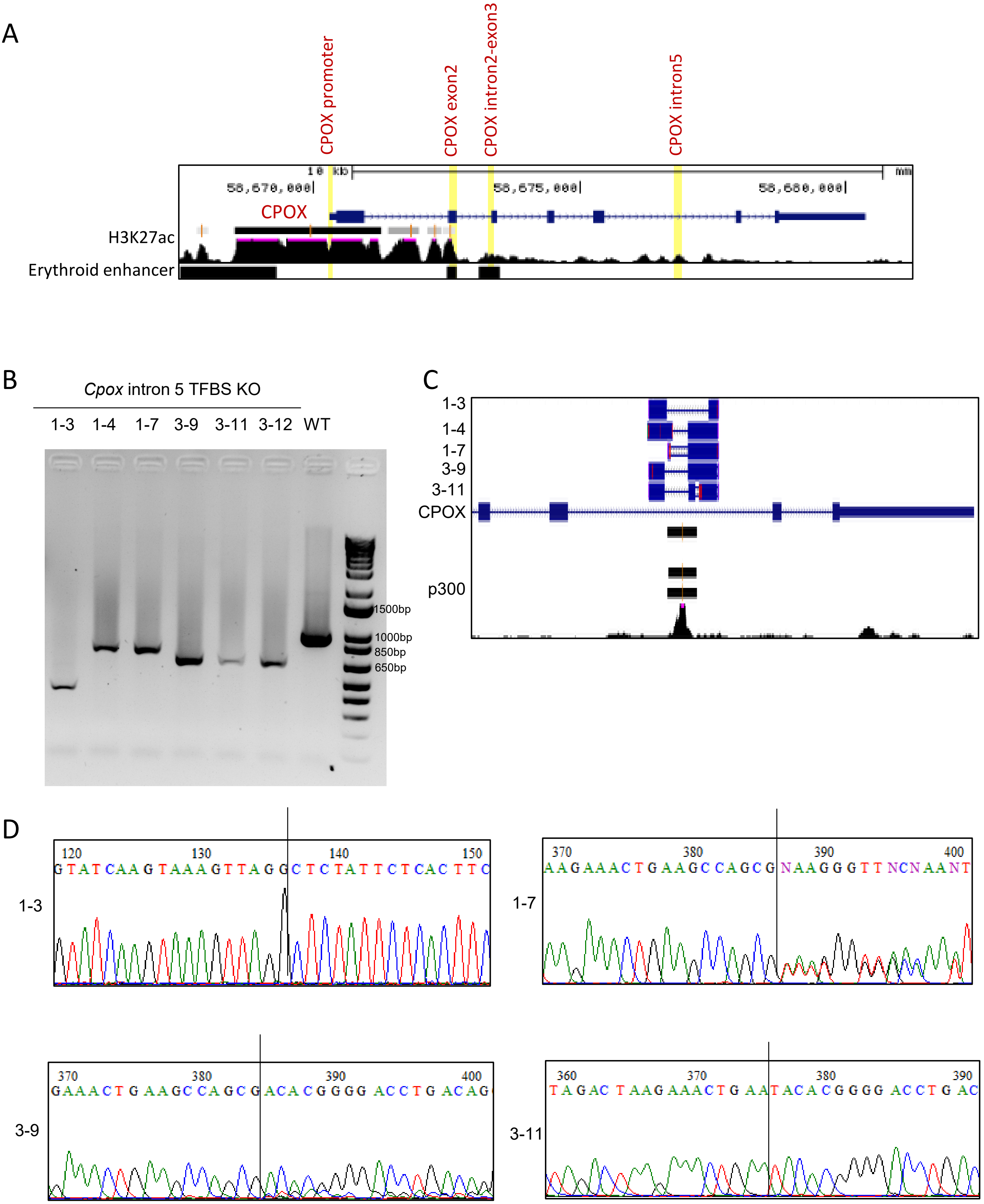

Supplementary Fig. 5

A

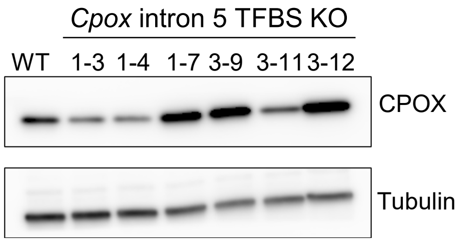

B

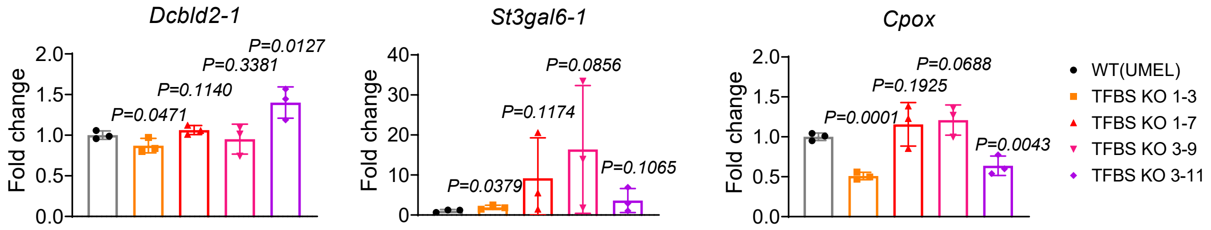

Supplementary Fig. 6

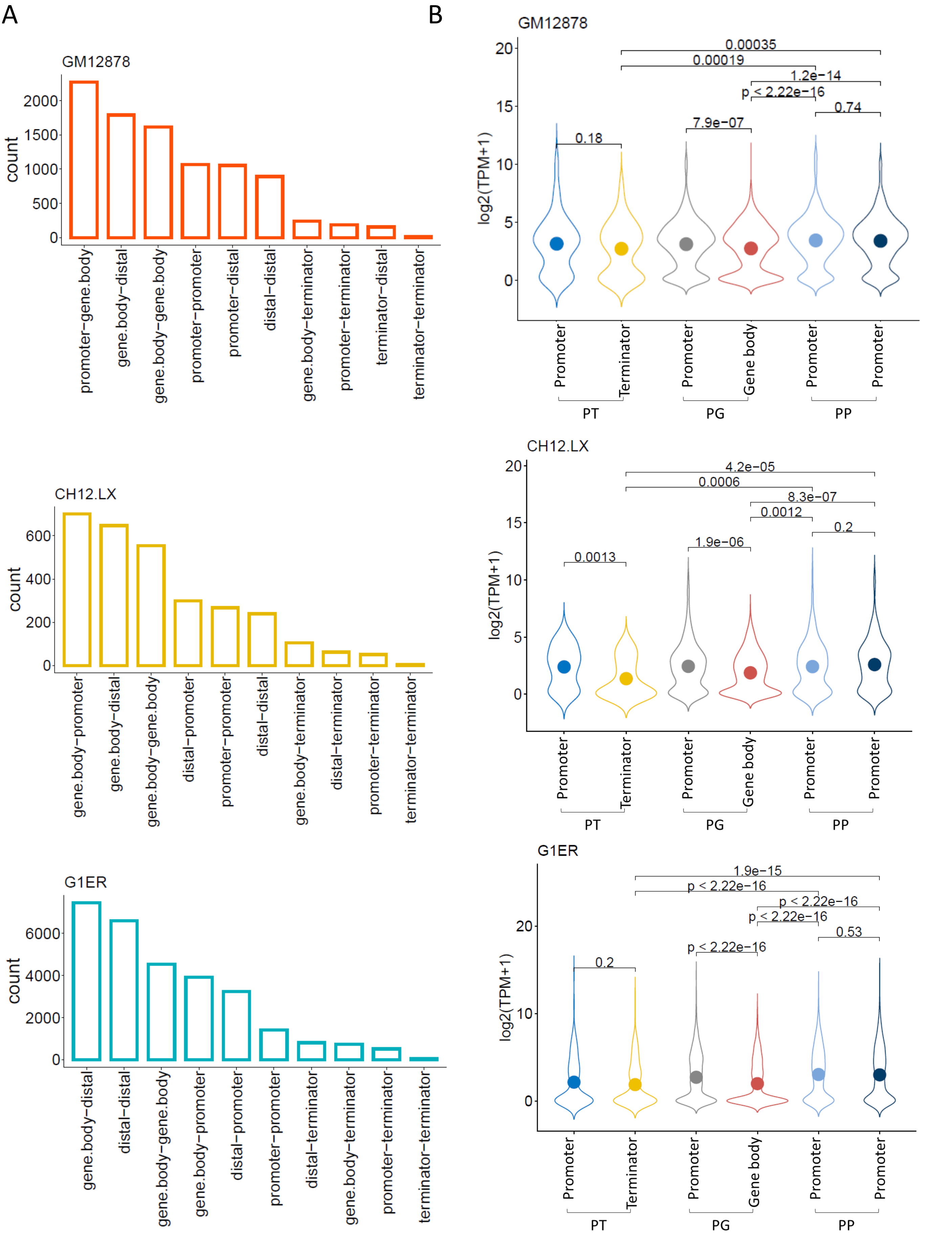

Supplementary Fig. 7

A

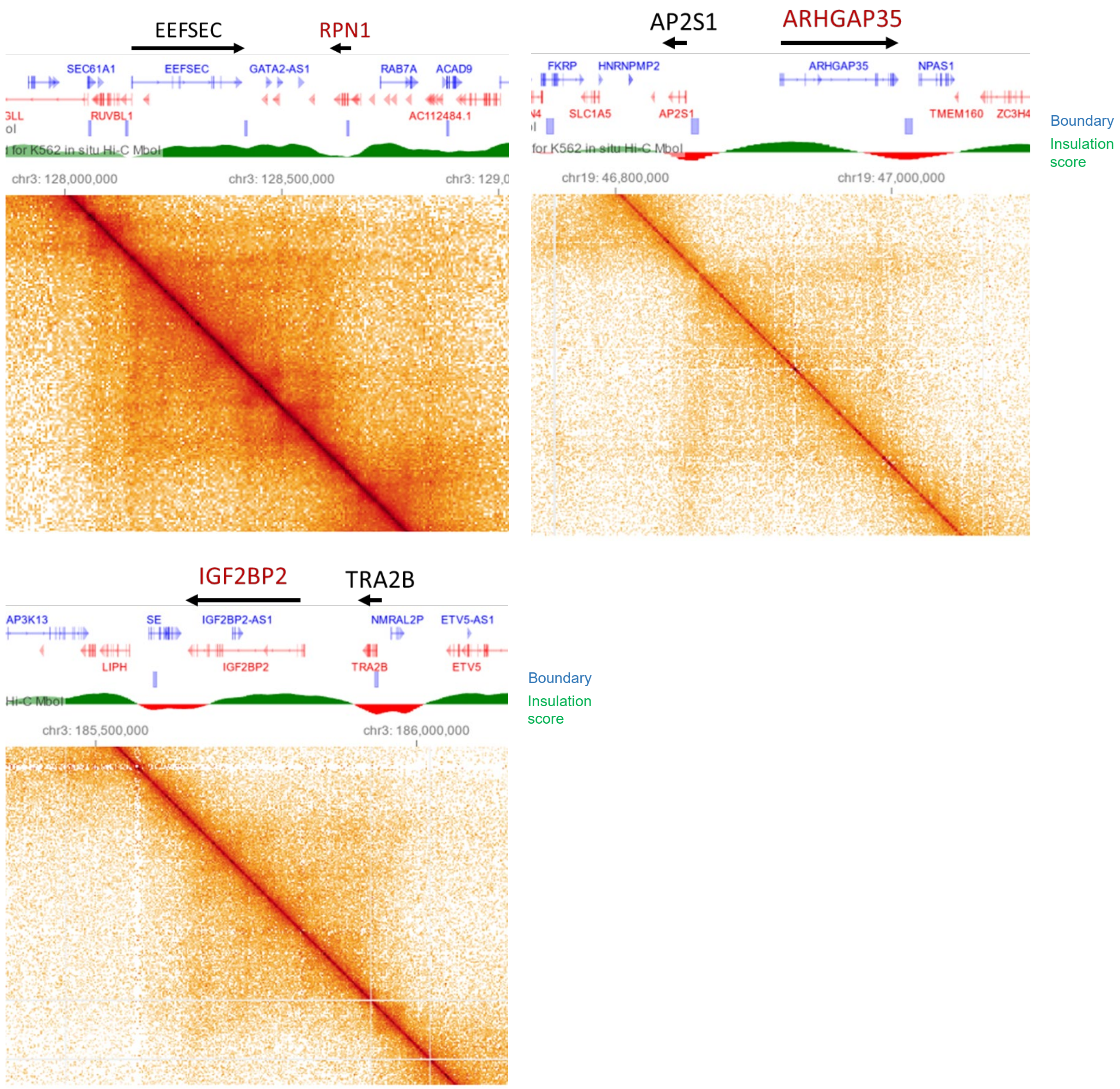

B

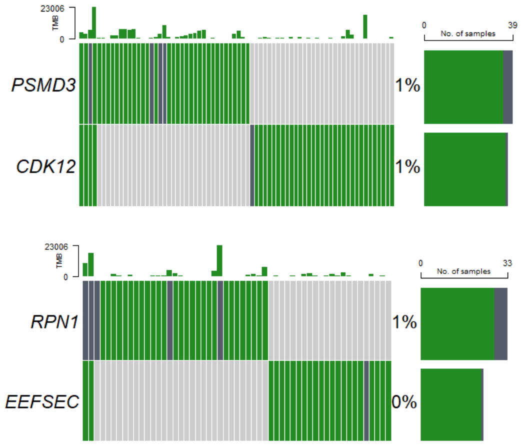

Supplement: 1 [file NIHPP2023.12.05.570115V1-supplement-1.pdf]
